# 高效液相色谱-二极管阵列检测法测定小麦粉及面粉改良剂中福美双

**DOI:** 10.3724/SP.J.1123.2020.07024

**Published:** 2021-06-08

**Authors:** Xuxin WANG, Shukun ZHOU, Xiaomin LI, Qinghe ZHANG

**Affiliations:** 中国计量科学研究院化学计量与分析科学研究所, 北京 100029; Division of Chemical Metrology and Analytical Science, National Institute of Metrology, Beijing 100029, China; 中国计量科学研究院化学计量与分析科学研究所, 北京 100029; Division of Chemical Metrology and Analytical Science, National Institute of Metrology, Beijing 100029, China; 中国计量科学研究院化学计量与分析科学研究所, 北京 100029; Division of Chemical Metrology and Analytical Science, National Institute of Metrology, Beijing 100029, China; 中国计量科学研究院化学计量与分析科学研究所, 北京 100029; Division of Chemical Metrology and Analytical Science, National Institute of Metrology, Beijing 100029, China

**Keywords:** 高效液相色谱-二极管阵列检测, 福美双, 小麦粉, 面粉改良剂, high performance liquid chromatography-diode array detection (HPLC-DAD), thiram, wheat flour, flour improvers

## Abstract

福美双是重要的二硫代氨基甲酸酯(DTC)杀菌剂,在小麦中使用限量以1 mg/kg二硫化碳(CS_2_)计。目前我国相关检测方法是针对二硫代氨基甲酸酯一类的化合物,二硫代氨基甲酸酯通过与酸反应生成CS_2_,采用光谱法或色谱法测定CS_2_,间接实现二硫代氨基甲酸酯测定。该方法无法特异性实现对福美双的检测,因此开展小麦粉中福美双检测方法的研究具有重要意义。研究建立了高效液相色谱-二极管阵列检测(HPLC-DAD)测定小麦粉及面粉改良剂中福美双的分析方法。小麦粉及面粉改良剂样品用乙腈溶剂提取后,经涡旋、振荡、冰水浴超声和静置后取上清液过滤,供高效液相色谱测定。采用ZORBAX plus-C18色谱柱(150 mm×4.6 mm, 5 μm)分离,以水-乙腈为流动相洗脱分析,在波长280 nm下检测。实验优化了提取溶剂及其体积、振荡超声条件、色谱柱、检测波长、流动相等条件。该方法采用保留时间和紫外光谱图定性,外标法定量。该方法在线性范围内(0.30~30.0 μg/mL)线性关系良好,相关系数(*r*^2^)为0.99999。对小麦粉及面粉改良剂进行1.5、3.0、15 mg/kg 3个水平的加标回收试验,福美双的回收率为89.6%~98.3%,相对标准偏差为1.6%~3.9%(*n*=6)。方法的检出限和定量限分别为0.5 mg/kg和1.5 mg/kg。该方法采用溶剂提取,操作简单,分析时间短,特异性好,具有精密度高、重复性好、检出限低等特点,适用于小麦粉及面粉改良剂中福美双快速、准确的定量检测。

## Abstract (en)

Thiram is an important dithiocarbamate (DTC) fungicide. In the United States and the European Union, the limit range of thiram is 0.1-15 mg/kg in fruits and vegetables, but there is no specific limit for grains. The maximum residue limit (MRL) for wheat is 1 mg/kg (calculated as carbon disulfide, CS_2_) in the National Food Safety Standard (GB 2763-2019). At present, the relevant regulation methods in China are targeted at the detection of dithiocarbamates and are incapable of detecting thiram specifically. CS_2_ is produced by the reaction of dithiocarbamate and acid, and it is then determined by spectrophotometry or GC; this renders the quantification of dithiocarbamate indirect. HPLC and HPLC-MS/MS methods are also reported for the detection of thiram. Most of the literature focuses on the determination of thiram in vegetables, fruits, soil, etc. In these methods, thiram is converted into dimethyldithiocarbamate (DMD) anions in an alkaline buffer solution, and DMD can be determined by HPLC-UV or LC-MS. However, ziram can also be converted into the DMD anion under alkaline conditions. Therefore, thiram cannot be distinguished from ziram, and this may produce false-positive results. Research has shown that in the presence of sulfite, thiram is converted into a DMD-sulfite adduct, which can be a marker for the selective determination of thiram. Furthermore, thiram can be directly detected by HPLC and HPLC-MS/MS after extraction with dichloromethane, chloroform, hexane, cyclohexane, ethyl acetate, or methanol and clean-up by solid phase extraction in vegetables and fruits. However, until now, few studies have reported the determination of thiram in wheat flour and flour improvers. Therefore, it is of great importance to develop a method for thiram in wheat flour. In this study, an analytical method based on HPLC-DAD was developed for the determination of thiram in wheat flour and flour improvers. The wheat flour and flour improver samples were extracted using acetonitrile. After shaking for 15 min, the samples were ultrasonicated for 10 min in an ice-water bath. The supernatant was filtered before separation on a ZORBAX plus-C18 column (150 mm×4.6 mm, 5 μm). The samples were eluted with a water-acetonitrile solvent system and detected at 280 nm. In this research, the extraction solvent, extraction solvent volume, ultrasonic conditions, chromatographic column, determination wavelength, and mobile phase were optimized. The retention times and UV spectra were used for qualitative analysis, and the external standard method was used to quantify thiram. Stability tests of standard stock solutions, a series of standard solutions, and extraction solutions were also performed. The standard stock solutions could be stored for at least 21 d, and the series of standard solutions could be stored for 14 d under refrigeration at 4 ℃. The standard solution was either exposed to light at room temperature for 4 h or kept in dark at room temperature for 48 h, and no obvious degradation was observed. This revealed that thiram was stable in acetonitrile solution during our investigation. It was suggested that the extraction solution should be analyzed as soon as possible. The linear range was 0.30-30.0 μg/mL. The peak area of the analyte showed a good linear relationship with its corresponding concentration, and the correlation coefficient (*r*^2^) was 0.99999. When the spiked levels were 1.5, 3.0, and 15 mg/kg, the spiked recoveries of thiram were 89.6%-98.3%, with relative standard deviations of 1.6%-3.9% (*n*=6). The limits of determination and quantification for thiram were 0.5 mg/kg and 1.5 mg/kg, respectively. The results revealed that this method is simple, rapid, and specific, in addition to having high precision, good repeatability, and a low limit of detection. The method is thus suitable for the daily routine analysis of thiram in wheat flour and flour improvers.

福美双(thiram)是重要的二硫代氨基甲酸酯(DTC)杀菌剂,DTC包括二甲基二硫代氨基甲酸酯(DMDs)、乙撑二硫代氨基甲酸酯(EBDs)和丙撑二硫代氨基甲酸酯(PBDs)^[1]^。福美双是二甲基二硫代氨基甲酸的氧化产物,是一种无金属杀菌剂。福美双可用作种子保护剂,用于水果、蔬菜、观赏植物和草坪作物的叶面处理,以控制真菌病害的数量,并保护收获的作物在储存或运输过程中不变质^[2]^。美国环境保护署法规(EPA-HQ-OPP-2009-0431-0001)和欧盟(EC) No 396/2005(《欧盟植物源和动物源食品及饲料中的农药最大残留限量》)均对福美双进行了限量规定,涉及食品基体主要是水果和蔬菜,限量范围为0.1~15 mg/kg,对谷物没有给出明确的限量。按照国家标准GB 2763-2019,小麦中福美双残留物以二硫化碳(CS_2_)表示,最大残留量为1 mg/kg,折算成福美双含量约为1.5 mg/kg。目前我国相关的检测方法是针对二硫代氨基甲酸酯一类的化合物,无法特异性的实现对福美双的检测,因此开展小麦粉中福美双检测方法的研究有重要意义。

目前,文献报道蔬菜、水果、土壤等中福美双通过转换、提取、净化等前处理后,采用光谱法^[3]^、气相色谱法(GC)^[4,5]^、高效液相色谱法(HPLC)^[6,7,8]^和高效液相色谱-串联质谱法(HPLC-MS/MS)^[1,2,9-13]^等方法检测。二硫代氨基甲酸酯易受pH值、基质成分和温度的影响,在样品前处理过程中容易降解^[2]^,所以传统检测方法将二硫代氨基甲酸酯与酸反应生成CS_2_,通过分光光度法或气相色谱法(GC)等技术测定CS_2_含量,间接计算二硫代氨基甲酸酯的总量^[4,14]^。但这类方法耗时,灵敏度低,且无法直接得到福美双的含量。福美双在碱性缓冲水溶液中,定量转化为DMD阴离子,通过HPLC-UV^[6]^或LC-MS测定DMD,间接实现福美双含量的测定^[10]^。福美锌在碱性条件下也降解为DMD阴离子,产生假阳性信号,这导致福美双检测结果的准确性低^[14]^。有研究证明,二硫代氨基甲酸酯在亚硫酸盐存在的情况下,仅福美双定量转化为DMD-亚硫酸盐加合物,因此DMD-亚硫酸盐加合物可作为福美双检测的标志物^[1]^。文献也有通过二氯甲烷、氯仿、己烷、环己烷、乙酸乙酯或甲醇等有机溶剂从蔬菜、水果基体中提取福美双,经固相萃取等净化后,采用HPLC、HPLC-MS/MS直接检测^[15,16]^。目前在小麦粉及面粉改良剂中检测福美双的文献较少,以上方法无论从基体适用性还是方法特异性,都不能满足小麦粉及面粉改良剂中福美双的定量检测。

本研究采用乙腈直接溶剂提取,经振荡、超声等操作后使用液相色谱法检测小麦粉及面粉改良剂中的福美双。通过对提取方式、色谱条件及溶液稳定性等因素的优化和评估,建立了准确检测小麦粉及面粉改良剂中福美双的方法。

## 1 实验部分

### 1.1 仪器与试剂

LC-20AD XR液相色谱仪,配有DAD二极管阵列检测器(日本岛津公司); xp205型电子天平(精度为0.01 mg,瑞士Mettler Toledo公司); Vortex-Genie 2型涡旋混合器(美国Scientific Industries公司); KQ 3000 VDV型超声波清洗器(昆山市超声仪器有限公司); IKA HS 260 Basic型往复式振荡摇床(德国IKA公司); Milli-Q型超纯水发生器(美国Millipore公司);娃哈哈纯净水(娃哈哈有限公司);聚丙烯(GHP)有机滤膜(孔径0.22 μm)。

乙腈、甲醇、正己烷、丙酮(色谱级,德国Merck公司);二氯甲烷(色谱级,中国克莱曼公司);四氢呋喃(色谱级,美国Sigma-Aldrich公司);福美双标准品(CAS号:137-26-8,德国Dr. Ehrenstorfer公司)。小麦粉、面粉改良剂均为市售。

### 1.2 标准溶液的配制

准确称取福美双标准品10 mg(精确至0.01 mg),置于100 mL容量瓶中,用乙腈溶解并定容至刻度,充分摇匀,配制成100 μg/mL的标准储备液,于4 ℃冷藏保存。

分别准确移取0.15、0.5、1.5、5.0和15 mL的标准储备液,置于50 mL容量瓶中,用乙腈稀释至刻度,混匀,配制质量浓度为0.3、1.0、3.0、10和30 μg/mL的系列标准工作液,于4 ℃冷藏保存。

### 1.3 样品前处理

取小麦粉或面粉改良剂代表性样品约100 g,混匀,装入洁净容器中,干燥避光保存。

称取1 g试样(精确到0.01 g),置于50 mL具塞塑料离心管中,加入乙腈5 mL,涡旋1 min,振荡15 min后,冰水浴超声10 min,静置2 min(如浑浊,以6000 r/min高速离心2 min),取上清液,过0.22 μm有机相滤膜,供测定。

### 1.4 分析条件

色谱柱:ZORBAX plus-C18色谱柱(150 mm×4.6 mm, 5 μm);柱温:25 ℃;流动相:(A)水和(B)乙腈;流速:1.5 mL/min。梯度洗脱程序:0.1~5.0 min, 50%B; 5.0~5.5 min, 50%B~90%B; 5.5~6.5 min, 90%B; 6.5~7.0 min, 90%B ~ 50%B; 7.0~10.0 min, 50%B。流速:1.5 mL/min。进样量:20 μL;检测波长:280 nm。

## 2 结果与讨论

### 2.1 溶解液的选择

分别采用异丙醇、甲醇、丙酮、乙腈配制福美双母液。结果表明,丙酮的溶解性最佳;乙腈配制后经充分振荡后溶解;异丙醇、甲醇需充分振荡和超声后溶解。丙酮色谱吸收截止波长为330 nm,容易对分析物造成干扰,因此采用乙腈制备母液。

### 2.2 前处理条件优化

2.2.1 提取溶剂优化

福美双的极性较弱,正辛醇/水分配系数的对数(log *K*_ow_)为1.73,不溶于水,可溶于乙腈、丙酮、二氯甲烷和氯仿等有机试剂。因此,实验采用1 g样品,比较了8 mL的正己烷、二氯甲烷、四氢呋喃、丙酮、乙腈、甲醇等溶剂的提取效果(见图1)。采用正己烷提取时,提取回收率为0;采用四氢呋喃、二氯甲烷和甲醇时,回收率分别为52%~60%、52%~62%和4%~6%;采用丙酮时,回收率为75%~85%,但由于溶剂效应,福美双峰形较差;采用乙腈时,提取回收率为86%~94%。Ekroth等^[17]^采用乙酸乙酯和环己烷的混合物提取果蔬中福美双,样品平均回收率为40%~86%。Dinesh等^[7]^采用反相高效液相色谱法测定芒果和石榴中福美双等多种农药,样品前处理采用乙腈提取和PSA分散固相萃取,平均回收率分别大于87%和96%。江泽军等^[18]^采用样品经乙腈提取,PSA、C18吸附剂净化,水稻植株或稻壳中福美双的回收率为76%~99%。因此,福美双的提取效率与基体有较大的相关性,针对小麦粉及面粉改良剂,选择乙腈作为提取溶剂。

**图 1:**
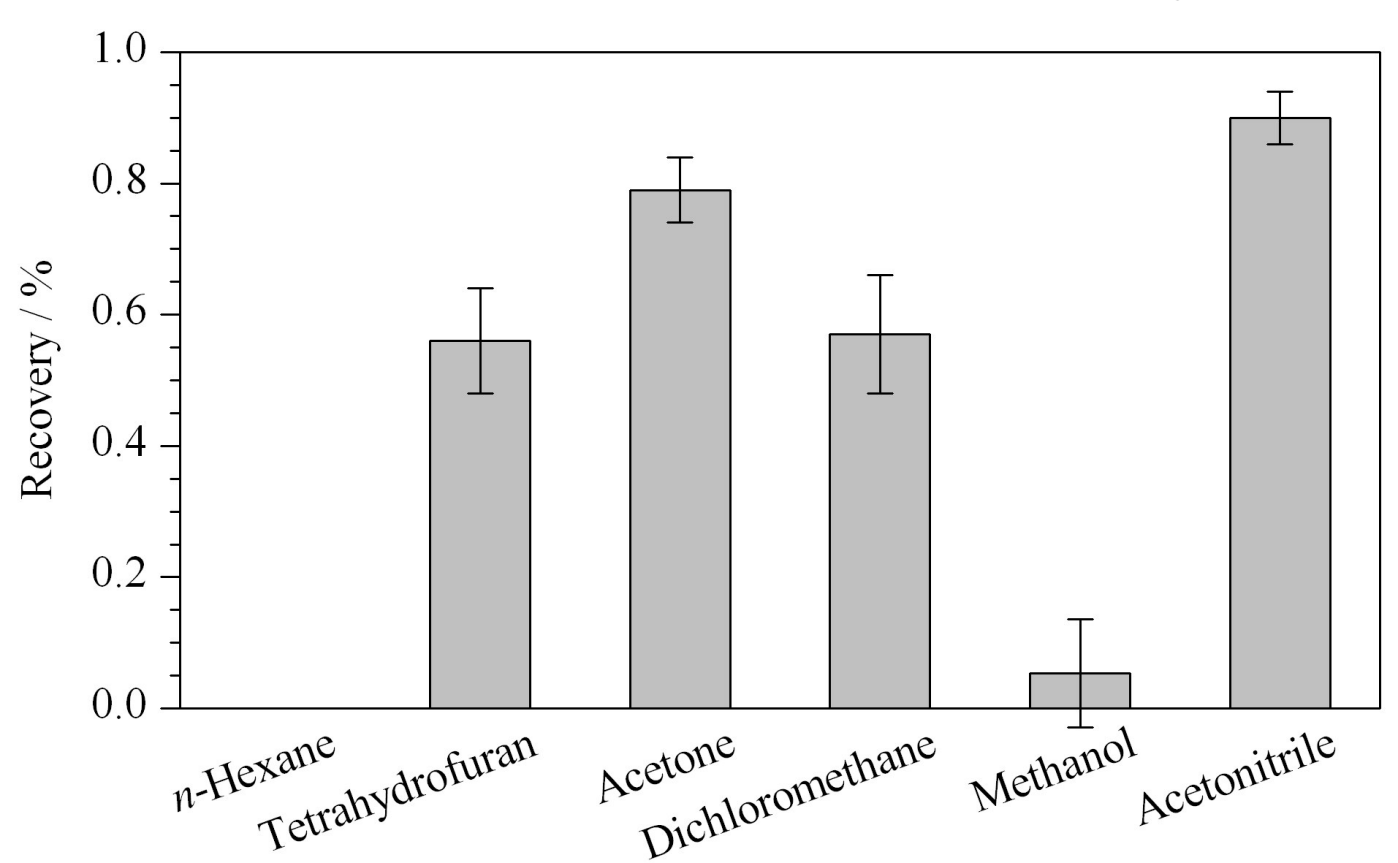
不同提取溶剂对福美双回收率的影响(*n*=3)

当样品加标水平为15 mg/kg,比较了1 g样品采用8 mL和5 mL乙腈提取时,福美双的回收率均为85%~110%,最终选用5 mL乙腈提取,满足灵敏度且节约溶剂。同时,比较了1 g样品5 mL乙腈提取和2 g样品10 mL乙腈提取,前者的回收率为100.8%, RSD为2.6%,后者回收率为95%, RSD为1.7%,两者没有明显差异。因此,采用1 g样品5 mL乙腈提取的前处理条件。

2.2.2 振荡、超声条件优化

实验比较了振荡与超声条件的影响。在加标水平为7.5 mg/kg和15 mg/kg时,振荡15 min,福美双的回收率均为85%~95%,振荡25 min时,回收率均为89%~100%。冰水浴超声10 min和15 min的条件,福美双的回收率均为92%~97%,没有明显差异。最终选择振荡15 min和冰水浴超声10 min的前处理条件。

### 2.3 液相色谱条件优化

2.3.1 色谱柱的选择

福美双分析主要有直接分析法和转化产物的间接分析法。福美双为弱极性化合物,直接法主要采用C18色谱柱^[2,12]^分析。间接法是在碱性条件下将福美双转化为DMD阴离子,采用亲水相互作用色谱柱(HILIC柱)检测^[19]^。

本实验比较了HILIC和Amide两种色谱柱直接检测福美双,结果显示福美双没有保留。另外本方法比较了BEH-C18 (50 mm×2.1 mm, 1.7 μm)、ZORBAX plus-C18 (150 mm×4.6 mm, 5 μm)和UNITARY C18 (150 mm×4.6 mm, 5 μm)色谱柱的分离情况。结果显示,3种色谱柱均能有效保留福美双。BEH-C18是超高效液相色谱柱,成本偏高,普适性不强;采用ZORBAX plus-C18色谱柱时,福美双的峰形较采用UNITARY C18色谱柱时对称性好,峰宽窄。因此,最终本研究采用ZORBAX plus-C18 (150 mm×4.6 mm, 5 μm)色谱柱。

2.3.2 检测波长的选择

采用二极管阵列检测器在200~400 nm扫描范围内,得到福美双的紫外吸收光谱图(见图2)。福美双在220、254和280 nm均有较强吸收,由于220 nm处溶剂干扰较大,因此比较福美双在254 nm和280 nm的检测特异性。在254 nm处,加标3.0 mg/kg时小麦粉(见图3a)和面粉改良剂(见图3b)基质干扰大于280 nm,不利于福美双的定量。因此选用280 nm为检测波长。

**图 2:**
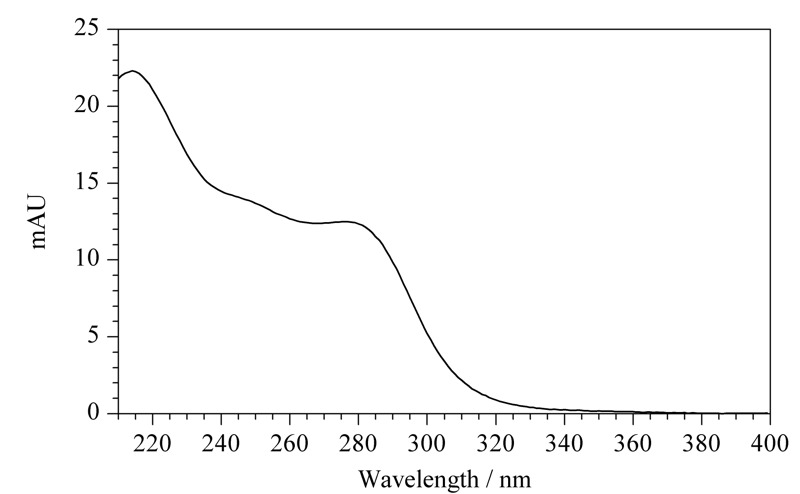
福美双的紫外吸收光谱图

**图 3:**
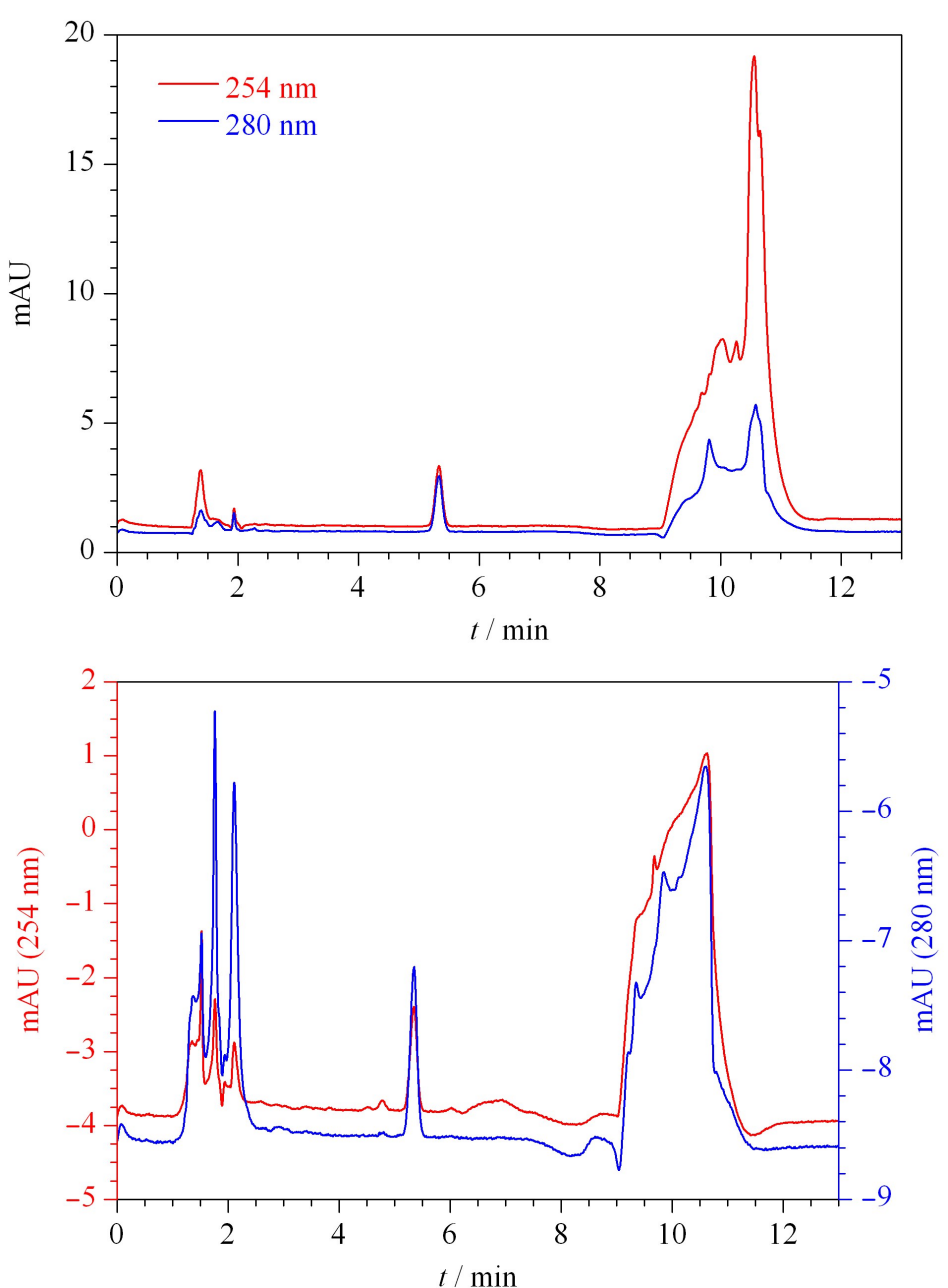
加标(a)小麦粉和(b)面粉改良剂中福美双(3.0 mg/kg)的色谱图

2.3.3 流动相的选择

福美双在碱性条件下生成DMD阴离子,在酸性条件下生成盐^[20]^,因此本研究不考虑添加酸或者缓冲体系,仅选择有机溶剂与水的分离条件。实验比较了水-甲醇和水-乙腈两种流动相体系。流动相为水-甲醇体系时,福美双无法与干扰成分有效分离(见图4a),因此考察水-乙腈体系。

**图 4:**
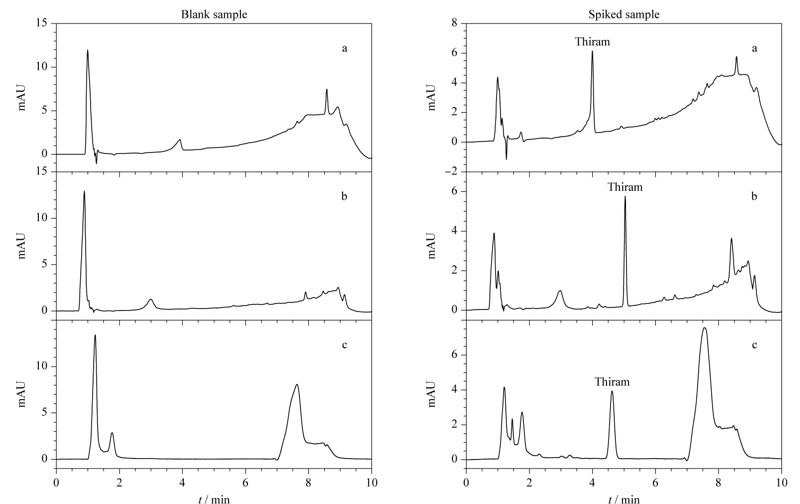
采用不同流动相和梯度洗脱程序时空白及加标样品的色谱图

采用水-乙腈梯度洗脱,杂质与福美双得到有效分离(见图4b),但是基线逐渐上升。考虑到低浓度检测的灵敏度,改变流动相梯度,福美双无干扰且基线稳定性得到改善(见图4c)。因此采用水-乙腈作为最终分离体系。Zhou等^[21]^采用C18色谱柱,流动相为乙腈-水(50∶50, v/v),能够完全分离食品样品中的福美双与其他物质。江泽军等^[18]^在水中添加了0.2%甲酸,并比较了甲醇-水和乙腈-水两种流动相体系,结果显示流动相为甲醇-水时,色谱柱压力较高,且基线噪声较大。

### 2.4 方法学验证

2.4.1 福美锌干扰分析

福美双和福美锌结构相似,且紫外光谱类似^[1]^,因此开展了福美锌干扰实验研究。比较了在小麦粉样品中分别添加10 mg/kg福美锌以及10 mg/kg福美锌、10 mg/kg福美双。结果显示,仅添加福美锌时,在福美双的色谱保留时间内没有色谱峰;同时添加福美锌和福美双时,福美双的回收率为92%~95%,说明福美锌的存在不干扰福美双的测定。

2.4.2 基质效应

文献中土壤和糙米对福美双具有基质抑制,应采用基质匹配校准溶液^[18]^,植株、稻壳、豆芽均无明显基质效应,可用溶剂校准溶液,采用外标法进行定量^[22]^。说明基质效应与基体密切相关,因此本方法比较了0.3、1.0和3.0 μg/mL由空白小麦粉经整个前处理过程得到的基质校准溶液和纯乙腈溶液的响应差异。结果显示,二者的响应没有差异,证明基质影响不显著。因此,直接采用溶剂配制标准溶液进行外标法定量。

2.4.3 线性范围、检出限和定量限

在0.30~30 μg/mL线性范围内,以福美双的质量浓度为横坐标(*x*, μg/mL)、峰面积为纵坐标(*y*)绘制标准曲线,得到线性回归方程为*y*=38.13*x*,相关系数(*r*^2^)为0.99999。

选择空白样品,定量添加标准溶液,当所得谱图的信噪比为3和10时,将添加量定义为检出限和定量限分别为0.5 mg/kg和1.5 mg/kg。果蔬等食品基质中福美双的定量限范围一般为0.01~2.5 mg/kg^[11,17,21-23]^。与文献相比,本研究采用直接检测,操作简单,灵敏度满足标准限量,可用于福美双的检测。

2.4.4 回收率与精密度

对一种小麦粉和两种面粉改良剂进行添加回收实验,添加水平分别为1.5、3.0和15 mg/kg。结果表明,福美双的平均加标回收率为89.6%~98.3%,相对标准偏差为1.6%~3.9%(*n*=6),方法精密度良好(见表1)。曹秀等^[24]^采用甲醇超声提取,超高效液相色谱-串联质谱法测定小麦粉中福美双残留量,加标回收率为70.52%~85.20%,相对标准偏差为8.74%~9.42%。结果显示,本研究方法有较高的回收率与精密度。

**表 1:** 小麦粉及面粉改良剂中福美双的加标回收率和精密度(*n*=6)

Sample	Added/(mg/kg)	Recovery/%	RSD/%
Wheat flour	1.5	93.3	3.9
	3.0	95.0	3.2
	15	96.9	1.9
Flour improver-1	1.5	89.6	3.1
	3.0	94.4	2.5
	15	98.3	1.6
Flour improver-2	1.5	93.5	2.4
	3.0	90.6	2.3
	15	97.7	1.7

### 2.5 稳定性

2.5.1 标准储备液稳定性

将福美双标准储备液(1.0 mg/mL)在4 ℃条件下棕色瓶密封保存,然后分别在1、2、14、21 d取出,稀释制成0.3、1.0、10.0 μg/mL的标准溶液,并将其与新制备的标准储备溶液比较。结果显示,储备液4 ℃放置21 d,福美双的峰面积与新配制的无显著性差异。因此,储备液4 ℃放置21 d稳定性良好。

2.5.2 系列标准溶液

将配制好的系列标准溶液(0.3、1.0、3.0、10.0和30.0 μg/mL)在4 ℃条件下棕色瓶密封保存,在一定时间后(2 d和14 d)取出测定。结果显示,0、2、14 d的系列标准溶液峰面积基本一致。因此,系列校准溶液在4 ℃冷藏避光下至少可以稳定保存14 d。

2.5.3 标准溶液的短期稳定性

取3 μg/mL福美双标准溶液置于棕色瓶中,分别考察室温光照4 h和室温避光48 h内福美双峰面积变化情况。福美双响应的相对标准偏差分别为1.5%和0.7%。可见福美双在乙腈溶液中较为稳定。

2.5.4 提取溶液的稳定性

在一种小麦粉样品、3种小麦粉改良剂样品中分别添加10 mg/kg福美双,将处理好的样品在5、12、24、36 h内连续进样,福美双在小麦粉和两种小麦粉改良剂中的响应变化均小于10.0%,而在一种小麦粉改良剂中的福美双响应下降50%。因此需要考虑提取液的稳定性,建议处理完12 h内上机测定。

## 3 结论

本文采用乙腈提取,HPLC-DAD直接测定小麦粉及面粉改良剂中福美双的方法。该方法操作简单,精密度高,灵敏度好,设备成本低,适用于实际大量检测使用。
